# Circulating antibody-secreting cells are a biomarker for early diagnosis in patients with Lyme disease

**DOI:** 10.1371/journal.pone.0293203

**Published:** 2023-11-03

**Authors:** Natalie S. Haddad, Sophia Nozick, Shant Ohanian, Robert Smith, Susan Elias, Paul G. Auwaerter, F. Eun-Hyung Lee, John L. Daiss

**Affiliations:** 1 MicroB-plex, Inc., Atlanta, GA, United States of America; 2 Division of Infectious Diseases, Maine Medical Center, MaineHealth Institute for Research, Portland, ME, United States of America; 3 Sherrilyn and Ken Fisher Center for Environmental Infectious Diseases, The Johns Hopkins School of Medicine, Baltimore, MD, United States of America; 4 Division of Pulmonary, Allergy & Immunology, Emory University, Atlanta, GA, United States of America; University of Minnesota, UNITED STATES

## Abstract

**Background:**

Diagnostic immunoassays for Lyme disease have several limitations including: 1) not all patients seroconvert; 2) seroconversion occurs later than symptom onset; and 3) serum antibody levels remain elevated long after resolution of the infection.

**Introduction:**

MENSA (**M**edium **E**nriched for **N**ewly **S**ynthesized **A**ntibodies) is a novel diagnostic fluid that contains antibodies produced in vitro by circulating antibody-secreting cells (ASC). It enables measurement of the active humoral immune response.

**Methods:**

In this observational, case-control study, we developed the MicroB-plex Anti-C6/Anti-pepC10 Immunoassay to measure antibodies specific for the *Borrelia burgdorferi* peptide antigens C6 and pepC10 and validated it using a CDC serum sample collection. Then we examined serum and MENSA samples from 36 uninfected Control subjects and 12 Newly Diagnosed Lyme Disease Patients.

**Results:**

Among the CDC samples, antibodies against C6 and/or pepC10 were detected in all seropositive Lyme patients (8/8), but not in sera from seronegative patients or healthy controls (0/24). Serum antibodies against C6 and pepC10 were detected in one of 36 uninfected control subjects (1/36); none were detected in the corresponding MENSA samples (0/36). In samples from newly diagnosed patients, serum antibodies identified 8/12 patients; MENSA antibodies also detected 8/12 patients. The two measures agreed on six positive individuals and differed on four others. In combination, the serum and MENSA tests identified 10/12 early Lyme patients. Typically, serum antibodies persisted 80 days or longer while MENSA antibodies declined to baseline within 40 days of successful treatment.

**Discussion:**

MENSA-based immunoassays present a promising complement to serum immunoassays for diagnosis and tracking therapeutic success in Lyme infections.

## Introduction

Lyme disease, caused by *Borrelia burgdorferi* infection, is transmitted by the bite of black-legged ticks of the genus Ixodes [1, 2]. It has become endemic in the northeastern and north-central United States, and the range expansion of infected ticks continues [3]. Diagnosis of early disease is based on the appearance of an erythema migrans rash (EM) that is typically centered on the tick bite and appears several days to three weeks later. In endemic regions, the EM rash and the awareness of a tick bite are sufficient to diagnose Lyme disease and initiate appropriate antibiotic therapy.

Supportive immunological diagnosis is based on the emergence of circulating antibodies specific to *B*. *burgdorferi*-associated protein antigens known to be immunogenic in healthy adults. These antibodies appear two-to-four weeks following the initiating tick bite and are typically measured by Two-Tier testing in the state-of-the art. In the first-tier, serum antibody responses against an extract containing *B*. *burgdorferi* antigens are measured in an enzyme immunoassay (EIA); but this single test is vulnerable to false positive results. Consequently, a second-tier test uses antigens in an immunoblot format so antibodies reactive with specific antigenic components can be recognized, thereby providing greater specificity [4]. Recent diagnostic improvements have included substitution of *B*. *burgdorferi* extracts with recombinant protein antigens and EIAs that test for antibodies specific for a characteristic peptide derived from a conserved segment of the variable major protein-like sequence E1 (VlsE1) identified as C6 [5, 6].

Collectively, these immunoassays remain the laboratory standard for Lyme diagnosis, but they have several limitations. Specifically, antibody levels are not detectable early in the infection; symptoms appear days or weeks before the emergence of circulating antibodies [7]. Second, these antibodies often remain elevated for months or years after the infection has been clinically resolved so they are considered unreliable indicators of disease resolution [8]. Finally, diagnoses of reinfection or recurrence are confounded by pre-existing antibodies [9–11].

**Fig 1:**
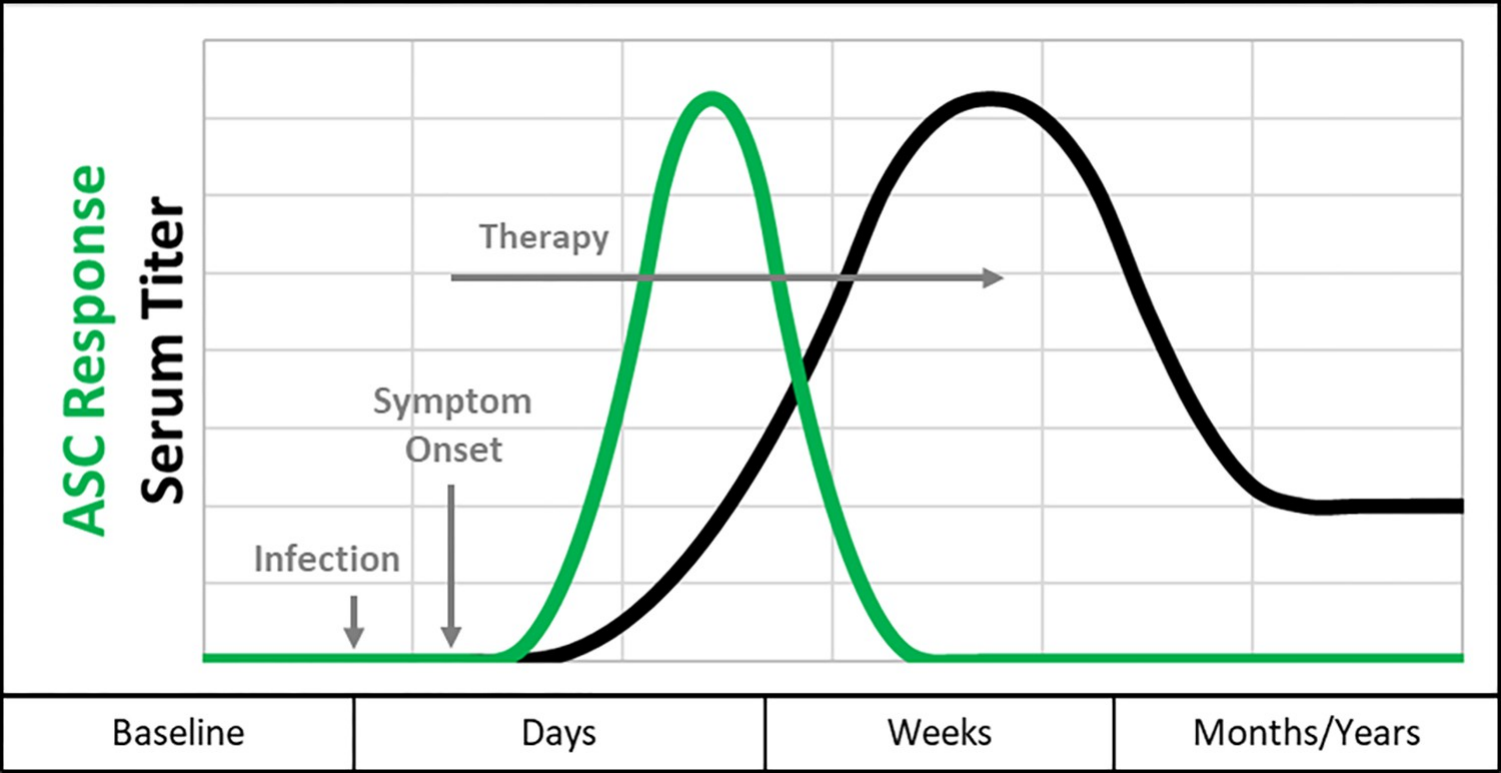
Kinetics of antibody-secreting cells compared to serum titer. At the start of primary infection, newly stimulated ASCs begin circulating, reach a peak response within a few days, then decline in response to successful therapy (green line); Newly secreted ASC-derived antibodies can be measured in MENSA. The serum response rises more slowly than the MENSA response and can last long after the infection has resolved (black line). Unlike the serum titer that can remain elevated months or years following successful removal of the infection, ASC/MENSA antibody levels typically decline to zero shortly after the infection resolves.

With these issues in mind, we postulated that the measurement of antibodies produced by circulating antibody secreting cells (ASC) might overcome some of the limitations of conventional, serum-based immunoassays. ASC appear in the blood shortly following the initiation of an infection and they decline rapidly when an infection has resolved, hence, ASC are plausible biomarkers for detection of an active infection (Fig 1) [12–17]. Measurement of the specific antibodies that ASC secrete requires: 1) collection of the ASC-containing peripheral blood mononuclear cells (PBMC) from a whole blood sample; 2) removal of potentially interfering serum antibodies; and 3) time in culture for the secretion of measurable quantities of ASC-derived antibodies (24 hours in current practice). The resulting culture fluid populated by the ASC-derived antibodies is called Medium Enriched for Newly Synthesized Antibodies (MENSA). The abundance of the new antibodies can be readily measured in high sensitivity immunoassay formats such as Luminex [18, 19].

In this preliminary study, we address fundamental analytic points essential for the construction of a tool with both clinical and laboratory utility. We show that in patients experiencing new *B*. *burgdorferi* infections, ASC are generated in sufficient numbers that their antibodies secreted in vitro can be readily measured in MENSA. Critically, non-infected subjects do not generate appreciable levels of anti-*B*. *burgdorferi* antibodies in their MENSA samples. Finally, levels of ASC-derived, *B*. *burgdorferi*-specific antibodies are high at the beginning of an infection and rapidly decline upon successful disease resolution or successful therapy.

## Materials and methods

### Overview of samples utilized and collected for this study

This study involved three separate collections of samples. 1) **CDC Lyme Serum Panel I**: **a standard reference collection for validation (n = 32)**: a sample set developed by the CDC to assist in the early stages of assay development; these samples are serum only and were used to validate the assays and antigens. 2) **Controls (n = 36)**: matched serum and MENSA samples prepared from uninfected subjects from endemic (n = 5) and non-endemic (n = 31) regions were collected and analyzed to set C_0_ values. 3) **Newly Diagnosed Lyme Disease Patients (n = 12)**: this sample collection consisted of matched serum and MENSA samples prepared from Newly Diagnosed Lyme Disease Patients; for eight of the twelve patients follow-up samples were obtained as late as 120 days post-diagnosis. Details on each sample group are provided below.

#### CDC Lyme Serum Panel I

The CDC Lyme Serum Panel I Sample Collection was obtained from the Centers for Disease Control in Ft. Collins, CO [20]. It consisted of 32 samples including: a) eight Two-Tier seropositive samples from patients with established Lyme disease (convalescent (n = 4), neurologic Lyme (n = 2) or Lyme arthritis (n = 2)); and b) 24 samples from Two-Tier seronegative subjects including early acute Lyme-infected patients (n = 4); patients with similarly presenting medical conditions (n = 12) and healthy subjects from endemic (n = 4) and non-endemic areas (n = 4). Serum samples were sent to MicroB-plex along with corresponding Two-Tier serology results performed by the CDC prior to shipment.

#### Enrollment of Newly Diagnosed Lyme Disease Patients

Patients over 18 years of age and newly diagnosed with *B*. *burgdorferi* infections (n = 12) were enrolled during the summers of 2016 and 2017 by physicians at a large primary care practice (InterMed, Portland, ME) under the direction of Dr. Robert Smith of the Maine Medical Center, Division of Infectious Diseases. Multiple blood draws, some as late as 120 days post symptom onset (DPSO), were obtained from eight of the twelve patients. During the summer of 2016, five Early Lyme patients were enrolled between July 19 and August 25; follow-up draws were collected up to December 5, 2016. In 2017, seven additional patients were enrolled between July 17 and October 11; the last follow-up draw was collected on January 3, 2018. Patients’ identities were known only to the recruiting physicians and their assistants in ongoing care. Samples were de-identified and labeled only with a sample code number along with relevant supporting data (age, race, sex, timing of tick bite, persistence of symptoms) prior to shipping to the MicroB-plex laboratory in Atlanta, GA, for analysis.

#### Enrollment of Control subjects

Two groups of Control subjects (hereafter referred to as Controls) were enrolled. The first group consisted of subjects from a non-endemic area, Atlanta, GA, where adults with no known Lyme disease were recruited at Emory University (n = 31). This Non-Endemic Control group was enrolled between Nov. 30, 2016, and August 15, 2017. A second population comprised healthy adults who lived in endemic regions in southeastern Maine (n = 5). This Endemic Control group was enrolled between July 7, 2016 to August 22, 2016.

#### Ethics statement

Written Informed Consent was obtained from each subject/patient and witnessed by the recruiter or physician prior to enrollment and sample/data collection. For samples collected in Maine, Protocol, Informed Consent documents, and sampling procedures were approved by the Maine Medical Center Institutional Review Board (#4852) for both Lyme-infected patients and non-infected controls. For Atlanta-area controls, Protocol and Informed Consent documents were approved by the Emory University Institutional Review Board (#60838).

### Sample collection and processing

From each of the 36 Control subjects and twelve Newly Diagnosed Lyme Disease Patients, 10–20 mL of whole blood was drawn in 10 mL, green-topped, heparinized tubes for MENSA. In addition, one red-topped, 4 mL tube was drawn for serum. Samples from Endemic Controls and Newly Diagnosed Lyme Disease Patients were placed at room temperature (RT) into insulated shipping containers designed to maintain temperatures between 15–35°C and shipped overnight for processing the following day in the MicroB-plex Laboratory in Atlanta, GA. Serum was prepared by removing the clot from the tube followed by gentle centrifugation (800 x g for 10 minutes) to remove residual cells and debris. Serum was collected, aliquoted and stored at -80°C for subsequent analysis.

#### Preparation of PBMC and MENSA

Peripheral blood mononuclear cells (PBMC) were prepared as previously reported [18]. Briefly, the ASC-containing PBMC population was isolated from the whole blood samples by centrifugation (800 x g for 25 min) using Lymphocyte Separation Media (Corning). The PBMC layer was carefully pipetted and transferred to a second tube, pelleted (800 x g for 10 minutes) and washed five times (800 x g for 5 minutes) with RPMI-1640 (Corning) to remove serum immunoglobulins. Erythrocyte lysis (5 mL; 5 min) was carried out using Gey’s solution (0.83% NH_4_Cl + 0.1% KHCO_3_ in dH_2_O, pH 7.0) after the second wash and cells were counted after the fourth. Harvested and washed PBMC were then plated at 10^6^ cells/mL (1 mL per well in a 12 well tissue culture plate) in R10 media (RPMI-1640 supplemented with 10% fetal bovine serum and 1% antibiotic/antimycotic; Gibco) and cultured for 24 hours at 37°C in a laboratory incubator supplemented with 5% CO_2_. Culture fluid was collected and centrifuged (800 x g for 5 minutes) to remove PBMC and resulting supernatant (MENSA) was aliquoted and stored frozen at -80°C for subsequent analysis [18].

### Immunoassay methods

#### Synthesis of antigens

With the intention of using the C6 peptide identical to that used in the Immunetics® C6 Lyme ELISA™, the peptide MKKDDQIAAAMVLRGMAKDGQFALK-COOH (Molecular weight (MW) = 3076.1), a conserved segment of variable major-protein-like sequence 1 (VlsE1) from *B*. *garinii*, was selected [21–24]. The peptide pepC10 [23, 25] (PVVAESPKKP-COOH; MW = 1390.65), a highly conserved segment of the outer surface protein, OspC, of *B*. *burgdorferi* was selected as a second antigen [26]. Each peptide was custom-synthesized by Thermo-Fisher (Rockville, Ill.) with an N-terminal, biotinylated, 6-aminohexanoic acid residue to facilitate immobilization on streptavidin-coated MagAvidin beads, comparable to the ELISA assays cited above. Thermo-Fisher provided certificates of analysis documenting molecular weight and purity.

#### Immunetics® C6 Lyme ELISA™

The Immunetics® C6 Lyme ELISA™ was purchased from Immunetics (Norwood, MA) and used according to the instructions provided. This test measures combined IgM and IgG anti-C6 antibodies and produces a quantitative outcome measurable in a microtiter plate reader at 450 nm (A_450_). The A_450_ can then be used to calculate the Lyme Index Value (LIV): LIV exceeding 1.1 is considered positive; LIV below 0.9 is negative; and LIV between 0.9 and 1.1 is equivocal. It should be noted that a comparable, commercial immunoassay for anti-pepC10 was not utilized, consequently some validation data are presented only for anti-C6.

#### Coupling biotinylated peptides to Magplex-Avidin microspheres

Biotinylated C6 and pepC10 peptides were conjugated to avidin-coupled MagPlex-Avidin microspheres, paramagnetic microparticles color-coded into spectrally distinct regions, via a standard avidin coupling procedure (Luminex). MagPlex-Avidin microspheres were washed three times with PBS-BN blocking/storage buffer (PBS, 1% BSA, 0.05% sodium azide, pH 7.5) on a magnetic separator (2 min) then incubated for 30 minutes in the dark, RT, on an end-over-end rotator, in a suspension of PBS-BN and 1 μg/mL peptide. Conjugated microspheres were then washed twice, resuspended at 10^6^ beads/mL PBS-BN, and stored at 4°C.

#### MicroB-plex Anti-C6/Anti-pepC10 Immunoassay

The MicroB-plex immunoassay for anti-C6 (IgM+IgG) or anti-pepC10 (IgM+IgG) antibodies will be referred to as the MicroB-plex Anti-C6/Anti-pepC10 Immunoassay in this report. For these assays, serum samples were diluted 1:1000 unless noted otherwise; MENSA samples were assayed undiluted. Then, 50 μL of each sample was mixed with 50 μL of assay diluent (PBS with 1% BSA) containing MagAvidin beads (Luminex Corp., Austin TX) conjugated with the biotinylated C6 or pepC10 peptides. After incubation in a microtiter plate on a plate shaker (800 rpm) for 60 minutes, RT, the beads were washed with assay buffer (PBS, 1% BSA). Then, 100 μL of PE-conjugated goat anti-human IgM and IgG (SouthernBiotech, Birmingham, AL), 3 μg/mL in PBS-1% BSA, was added to the washed beads, and the mixture was incubated (800 rpm, RT, 30 minutes) before being washed again, resuspended in assay buffer, and read on a Luminex MagPix instrument with xPONENT software (Austin, TX). Median Fluorescent Intensity (MFI) results were corrected for background fluorescence levels (assay diluent for serum; R10 for MENSA) and reported as Median Fluorescent Intensity minus Background (MFI-B = net MFI).

### Data generation and analysis

All analyses on Luminex were performed using samples that had been stored at -80°C within 30 hours of collection (serum) or preparation (MENSA) and thawed only once for analysis. Samples were analyzed within six hours of thawing in batches in November 2016, August 2017 and February 2018. Standard samples provided by Immunetics and Zeus were used to ensure consistency of behavior in each assay format, including the MicroB-plex Anti-C6/Anti-pepC10 Immunoassay. Serum and MENSA MFI-B values were examined from the 31 non-endemic controls and five endemic controls to determine the C_0_ threshold of positivity. One non-endemic control exhibited serum C6 and pepC10 levels greater than 10 times the median value of all 36 controls and was thus eliminated from the C_0_ calculation. The average net MFI plus four standard deviations was calculated from the remaining 30 non-endemic and five endemic control subjects for both serum and MENSA C_0_ values. Definitive diagnosis of Lyme-infected patients was based on the presence of erythema migrans rash and/or associated with a recent tick bite; immune response was assessed using the Immunetics® C6 Lyme ELISA™; the variable under examination was the predictive value of the MicroB-plex Anti-C6/Anti-pepC10 Immunoassay. Further analyses, including unpaired t-tests and data processing, were carried out using Microsoft Excel and GraphPad Prism software.

#### Calculation of diagnostic sensitivity and specificity

Diagnostic sensitivity, or the ability of a diagnostic test to correctly identify disease or illness, was calculated as the proportion of true positive test results among individuals suffering the disease or illness. Diagnostic specificity, or the ability of a diagnostic test to correctly identify the absence of a disease or illness, was calculated as the proportion of true negative test results among individuals who do not have the disease or illness [27].

## Results

### Diagnostic sensitivity and specificity of Lyme disease immunoassays tested on a standard serum reference panel

**Fig 2:**
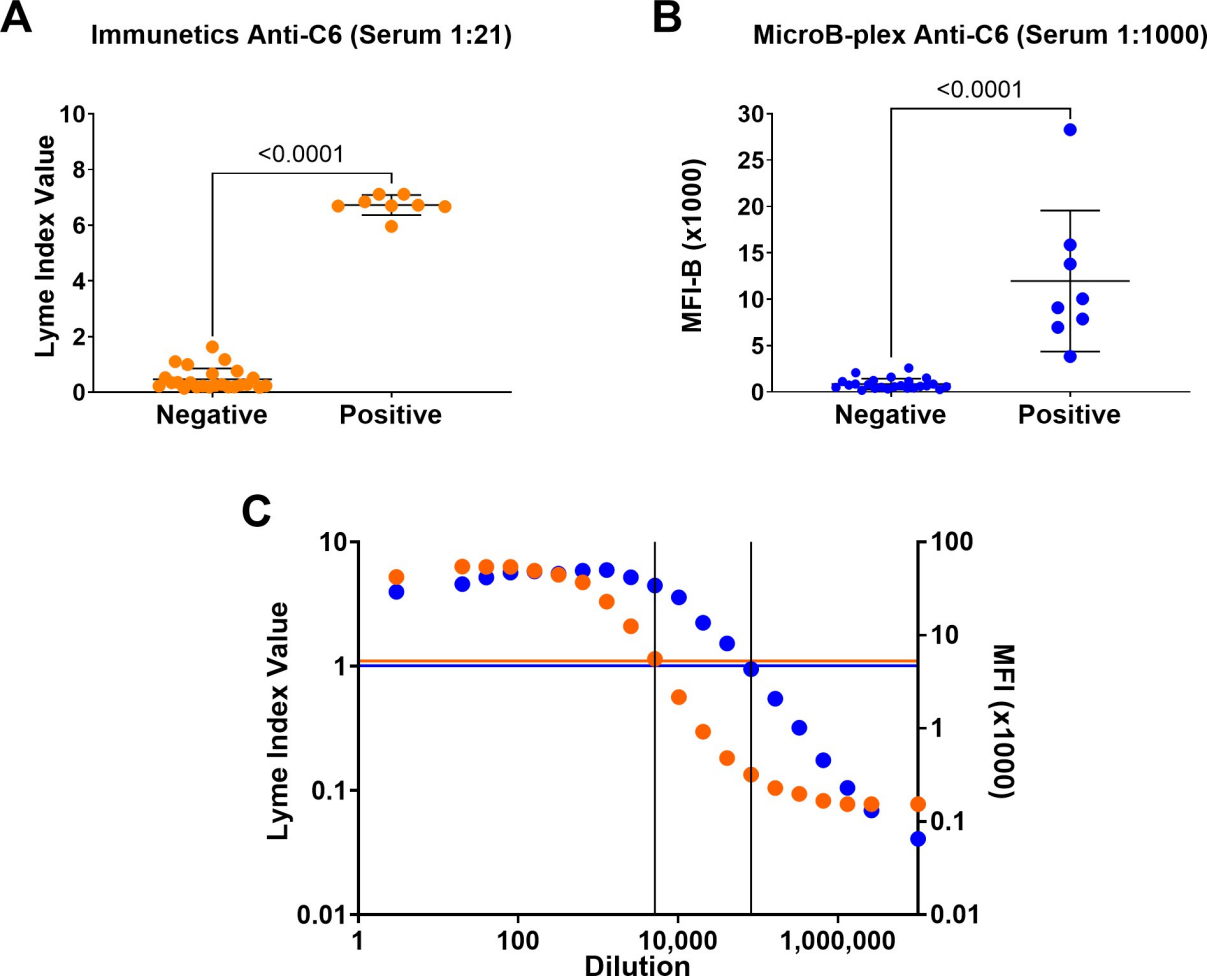
The MicroB-plex Anti-C6/Anti-pepC10 Immunoassay is concordant with the commercially available Immunetics® C6 Lyme ELISA™ and has greater analytic sensitivity. Anti-C6 levels were measured in the 32 serum samples from the CDC Lyme Serum Panel I. This collection included eight Two-Tier seropositive samples from patients with: i) convalescent Lyme disease or ii) neurologic Lyme disease or Lyme arthritis. In addition, there were 24 sera from Two-Tier seronegative patients who were: iii) healthy controls; iv) patients who had early acute Lyme (undetectable serum antibody); or v) patients who had potentially confounding medical conditions. Responses were measured using the Immunetics® C6 Lyme ELISA™ (orange dots) and the MicroB-plex Anti-C6/Anti-pepC10 Immunoassay (blue dots). Unpaired t-tests comparing the seronegative and seropositive populations were performed for each assay. A) The Immunetics® C6 Lyme ELISA™ produces a quantitative outcome called the Lyme Index Value. Seropositive patients and seronegative subjects from the CDC Lyme Serum Panel I were readily resolved using serum samples diluted 1:21 (p<0.0001). B) The same serum samples were resolved in the MicroB-plex Anti-C6/Anti-pepC10 Immunoassay using samples diluted 1:1000 (p<0.0001). C) Direct comparison of the MicroB-plex Anti-C6/Anti-pepC10 Immunoassay (blue) and the Immunetics® C6 Lyme ELISA™ (orange) using dilutions of serum from a single positive patient (IMD-EL-004). Orange horizonal line indicates the threshold for seropositivity (C_0_) in the Immunetics® C6 Lyme ELISA™; blue horizontal line indicates C_0_ for the MicroB-plex Anti-C6/Anti-pepC10 Immunoassay. Vertical lines indicate the serum dilutions at which the C_0_ was reached for each assay: 1:5,120 for the Immunetics® C6 Lyme ELISA™ and 1:81,920 for the MicroB-plex Anti-C6/Anti-pepC10 Immunoassay.

The diagnostic sensitivities and specificities of the MicroB-plex Anti-C6/Anti-pepC10 Immunoassay, and the Immunetics® C6 Lyme ELISA™, a commercially available in vitro diagnostic (IVD) ELISA assay, were assessed by comparison to the reference immunoassay (the CDC Two-Tier serology test) using the CDC Lyme Serum Panel I (Table 1). The CDC Two-Tier test resolved the population into two distinct groups: 1) Eight seropositives who included convalescent Lyme patients (n = 4) and prior Lyme patients suffering long-term sequelae (n = 2 neurologic Lyme, n = 2 Lyme arthritis); and 2) twenty-four seronegatives who were healthy subjects (n = 4 endemic, n = 4 non-endemic), had early acute Lyme infections (n = 4), or had potentially confounding, non-Lyme medical conditions (n = 12). Tested against the serum samples diluted 1:21, the Immunetics® C6 Lyme ELISA™ was concordant with the CDC Two-Tier test (Fig 2A and Table 1; 100% sensitivity and specificity). The MicroB-plex Anti-C6/Anti-pepC10 Immunoassay yielded similar results, even when the serum was diluted 1:1000 (Fig 2B and Table 1; 100% sensitivity and specificity). Unpaired t-tests comparing the seronegative and seropositive populations yielded highly significant p-values (p<0.0001) for both the Immunetics® C6 Lyme ELISA™ (Fig 2A) and the MicroB-plex Anti-C6/Anti-pepC10 Immunoassay (Fig 2B). In assessing the ability to diagnose Lyme disease at any stage (acute, convalescent, and post-Lyme sequelae), all three tests performed identically (Table 1). Each test identified patients who had prior Lyme Disease, whether they had ongoing sequelae (n = 4) or not (n = 4), but none of the three immunoassay methods was able to identify patients with acute early Lyme disease (n = 4). Thus, the diagnostic sensitivity for each test in this sample population was 67%. In terms of diagnostic specificity, all three tests yielded negative results for the 20 non-Lyme control subjects (100%).

**Table 1:** Comparison of diagnostic sensitivities and specificities of the standard Two-Tier test, the Immunetics® C6 Lyme ELISA™, and the MicroB-plex Anti-C6 Immunoassay using the 32 sample reference set CDC Lyme Serum Panel I.

Measure	Standard Two-Tier Test*	Immunetics Anti-C6 (Serum 1:21)	MicroB-plex Anti-C6 (Serum 1:1000)
**Concordance with Standard Two-Tier Serology Results**			
**Sensitivity**	100% (8/8)	100% (8/8)	100% (8/8)
**Specificity**	100% (24/24)	100% (24/24)	100% (24/24)
**Identification of Past or Present Lyme Disease**			
**Sensitivity**	67% (8/12)	67% (8/12)	67% (8/12)
**Specificity**	100% (20/20)	100% (20/20)	100% (20/20)

* Reference assay performed by CDC

### Analytic sensitivity of the MicroB-plex Anti-C6/Anti-pepC10 Immunoassay

MENSA samples typically have antibody concentrations several orders of magnitude lower than those measured in serum. Preliminary experiments using the Immunetics® C6 Lyme ELISA™ yielded consistently negative results on MENSA samples (data not shown). A direct comparison of the analytic sensitivity of the two immunoassay formats is illustrated by the titration of a single, positive serum sample (Fig 2C). Positive responses were detected at a 16-fold larger sample dilution in the MicroB-plex Anti-C6/Anti-pepC10 Immunoassay relative to the Immunetics® C6 Lyme ELISA™.

### Enrolled subjects: Controls and Newly Diagnosed Lyme Disease Patients

To test the potential utility of MENSA for Lyme disease diagnostics, we enrolled Control and Newly Diagnosed Lyme Disease Patient populations. Briefly, the endemic Control (n = 5) and Newly Diagnosed Lyme Disease Patient (n = 12) populations were predominantly male, exclusively white, with average ages in the late and early fifties, respectively (Table 2). The non-endemic Control population (n = 31) was more diverse in race (58% black) and sex (52% female) reflecting the regional demographics of Atlanta, GA. It also trended younger: 42.8 years of age compared to 53.8 years for the combined, Maine-based endemic Control and Newly Diagnosed Lyme Disease Patient populations.

**Table 2:** Demographics of enrolled populations.

Enrolled Group	Number	Sex (%)	Race n (%)	Age (S.D.)
**Non-endemic Controls (Georgia)**	31	Male 15 (48)	White 8 (26)	42.8 (12.5)
		Female 16 (52)	Black 17 (55)	
			Other 6 (19)	
**Endemic Controls (Maine)**	5	Male 5 (100)	White 5 (100)	58 (15.6)
**Newly Diagnosed Lyme Disease Patients (Maine)**	12	Male 9 (75)	White 12 (100)	52.1 (15.9)
		Female 3 (25)		

### Establishing cut-off (C_0_) values: Peptide antigens C6 and pepC10 were recognized by antibodies in serum and MENSA samples from Newly Diagnosed Lyme Disease Patients but not from Control subjects

Serum and MENSA samples collected from the 36 non-infected Control subjects were evaluated using the MicroB-plex Anti-C6/Anti-pepC10 Immunoassay. One non-endemic control exhibited serum C6 and pepC10 levels greater than 10 times the median value of all 36 controls, suggestive of prior infection, and was consequently eliminated from the C_0_ threshold of positivity calculation (Gray dot in S1A and S1B Fig). Analysis of serum responses from the remaining 30 non-endemic and five endemic Control subjects yielded C_0_ values set at the means plus four standard deviations (4,692 MFI-B for anti-C6 and 2,267 MFI-B for anti-pepC10; S1A and S1B Fig). The C_0_ values were set at the means plus four standard deviations in MENSA as well (29 MFI-B for anti-C6 and 26 MFI-B for anti-pepC10, S1C and S1D Fig). These C_0_ levels were clearly exceeded in MENSA and serum samples from two recently infected patients (Acute Lyme samples in S1A–S1D Fig).

### MENSA and serum anti-C6 and anti-pepC10 antibody levels are significantly higher in the Newly Diagnosed Lyme Disease Patient population than in the Control population

**Fig 3:**
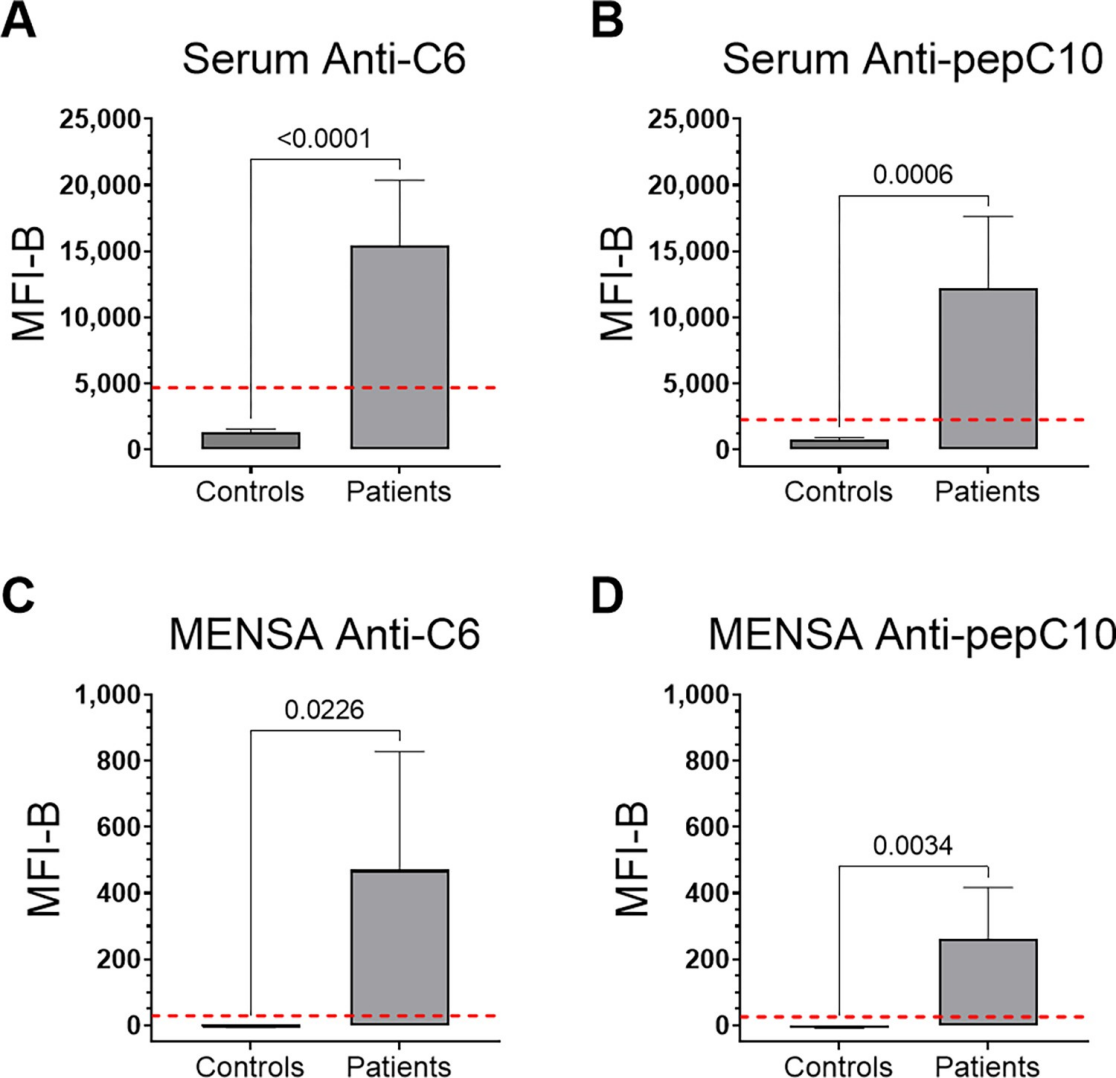
The Newly Diagnosed Lyme Disease Patient population has significantly higher MENSA and serum anti-C6 and anti-pepC10 antibody levels than the Control population. Average net MFI values were calculated for A) serum anti-C6, B) serum anti-pepC10, C) MENSA anti-C6, and D) MENSA anti-pepC10, for the Newly Diagnosed Lyme Disease Patient population (n = 12) between 6–18 DPSO and compared to the average net MFI values of the Control population (n = 36). The p-values are indicated above the pairwise comparison brackets. Red dashed lines indicate the C0 thresholds of positivity for each panel.

The Newly Diagnosed Lyme Disease Patient MENSA and serum samples obtained during 6–18 DPSO were assessed for anti-C6 and anti-pepC10 antibody levels. Unpaired t-tests comparing the average net MFI values of the twelve Newly Diagnosed Lyme Disease Patients against the 36 Control subjects yielded significant p-values for all comparisons (serum and MENSA, anti-C6 and anti-pepC10; p<0.0001 to p = 0.0226; Fig 3).

### Diagnostic sensitivity and specificity of the MicroB-plex Anti-C6/Anti-pepC10 Immunoassay comparing MENSA and serum samples

**Fig 4:**
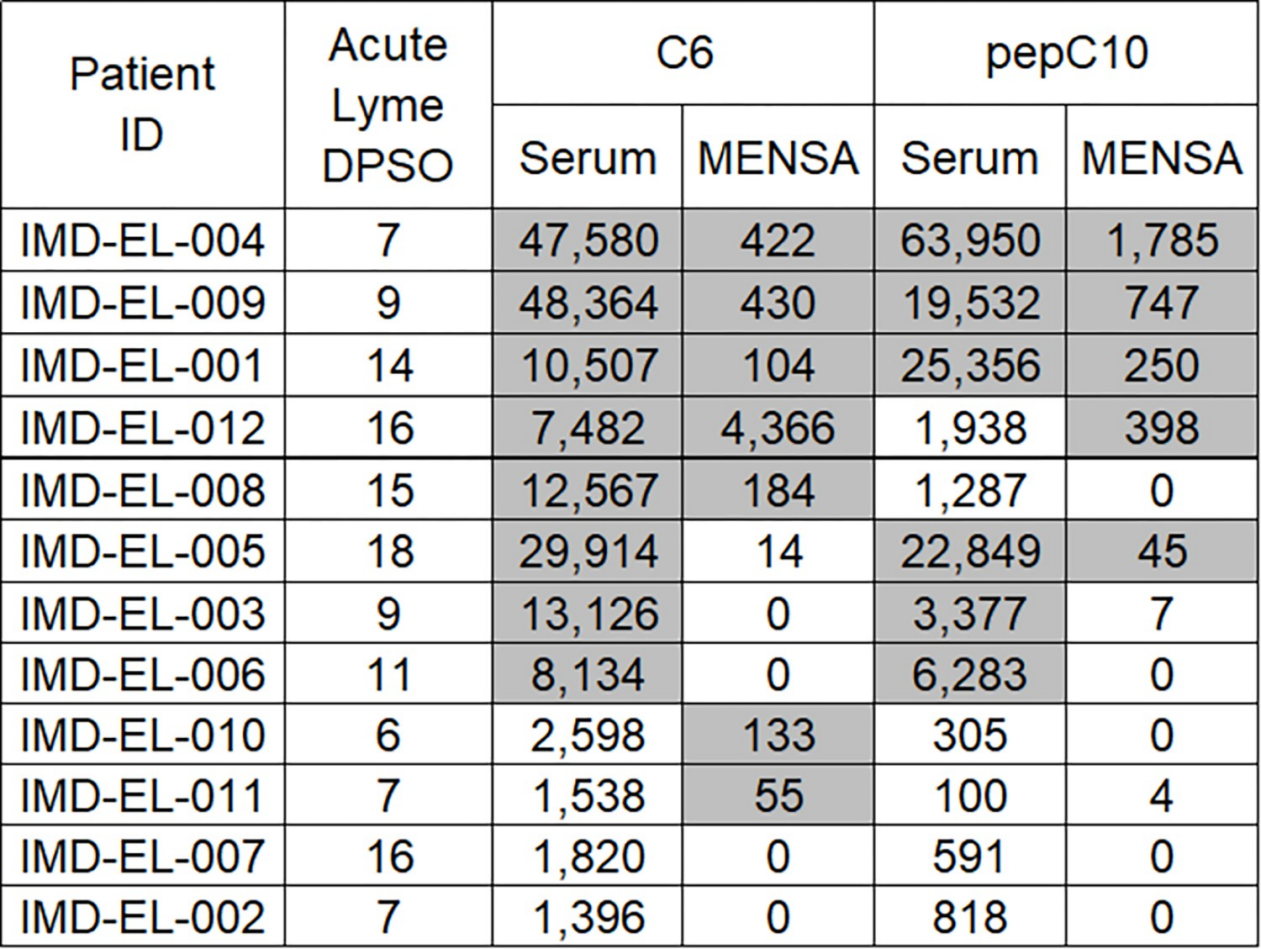
Measurement of anti-C6 or anti-pepC10 antibodies yields comparable results for diagnosis of early infections in serum and MENSA samples from Newly Diagnosed Lyme Disease Patients. Serum and MENSA antibodies specific for C6 and pepC10 were measured using the MicroB-plex Anti-C6/anti-pepC10 Immunoassay in samples from 12 Newly Diagnosed Lyme Disease Patients enrolled with suspected Lyme infections. Samples were collected at a single time point for each patient during the first 6–18 DPSO. For each patient, serum and MENSA antibody responses to C6 and pepC10 are presented as MFI-B values in a single horizontal row, with the corresponding patient identification number and DPSO of sample collection listed to the left. Positive response values are indicated as grey-shaded boxes; negative values are indicated as unshaded white boxes.

Results for each individual Newly Diagnosed Lyme Disease Patient at 6–18 DPSO are presented in Fig 4, where they are arranged by reactivity pattern. The first six patients (IMD-EL-004 to IMD-EL-005) were positive in MENSA and serum samples for anti-C6 and/or anti-pepC10. Two patients (IMD-EL-003, IMD-EL-006) were positive only in their serum samples while two others (IMD-EL-010, IMD-EL-011) were positive only in their MENSA samples. Two patients (IMD-EL-007, IMD-EL-002) were negative by all four measures. Eight patients were seropositive for anti-C6 by the MicroB-plex Anti-C6/Anti-pepC10 Immunoassay and six were seropositive for anti-pepC10. The six serum samples positive for anti-pepC10 were also positive for anti-C6. Among the MENSA samples, seven patients were positive for anti-C6 and five were positive for anti-pepC10; in combination, eight patients were positive for one or both peptide antigens in the MENSA samples (Table 3). Overall, serum anti-C6 was positive in eight of the ten patients who were positive by any measure while MENSA anti-C6 was positive in seven patients and one more was positive for anti-pepC10. Each sample type was positive for two patients who had scored negative in the other. Combining serum and MENSA results, the diagnostic sensitivity rises from 67% (8/12) to 83% (10/12). Using the Control population, all MENSA antigen combinations yielded 100% specificity (36/36, Table 3, S1C and S1D Fig). Since the non-endemic control subject eliminated from the C_0_ calculation above had anti-C6 and anti-pepC10 serum antibody levels above the C_0_ thresholds, the specificity for all serum antigen combinations was 97% (35/36, Table 3 and S1A and S1B Fig).

**Table 3:** Diagnostic sensitivities and specificities of MicroB-plex Anti-C6/Anti-pepC10 Immunoassay in MENSA and serum.

Test Combination	C6	pepC10	C6 and/or pepC10
**Serum**			
**Sensitivity**	67% (8/12)	50% (6/12)	67% (8/12)
**Specificity**	97% (35/36)	97% (35/36)	97% (35/36)
**MENSA**			
**Sensitivity**	58% (7/12)	42% (5/12)	67% (8/12)
**Specificity**	100% (36/36)	100% (36/36)	100% (36/36)
**Serum or MENSA**			
**Sensitivity**	83% (10/12)	58% (7/12)	83% (10/12)
**Specificity**	97% (35/36)	97% (35/36)	97% (35/36)

### The Newly Diagnosed Lyme Disease Patient population displayed multiple types of responses through the first 120 DPSO

**Fig 5:**
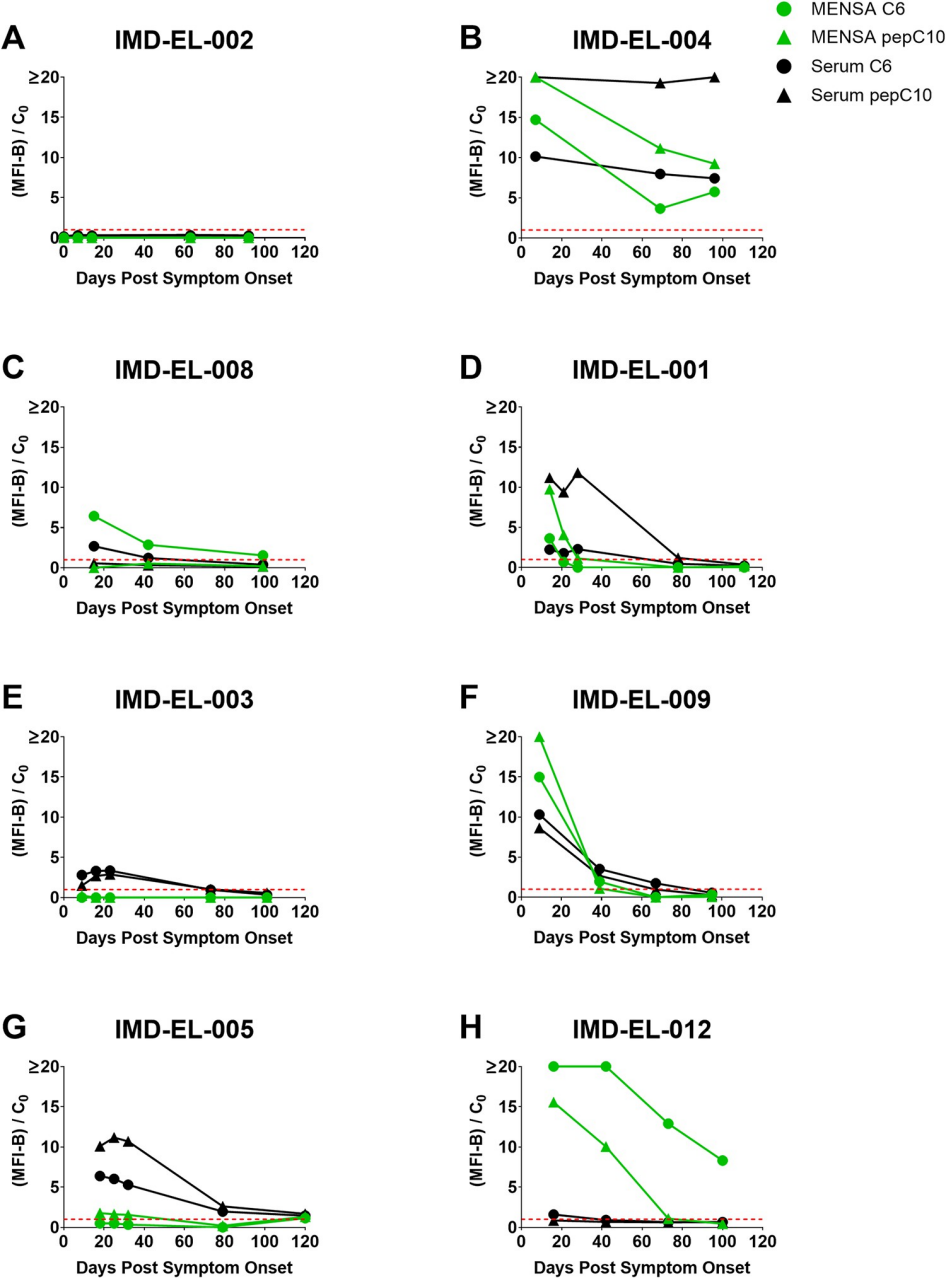
Multiple serum and MENSA response patterns were observed over the first 120 DPSO. Eight of the twelve enrolled Newly Diagnosed Lyme Disease Patients provided multiple samples during the period between 0 and 120 DPSO. Antibody responses from the MicroB-plex Anti-C6/anti-pepC10 Immunoassay are presented in panels A-H. Each patient’s identification number is listed at the top of the panel. To simplify data presentation, antibody levels specific for either C6 (circles) or pepC10 (triangles) are presented as MFI-B divided by their respective C_0_ values; horizontal red dashed line is set at 1 to represent the threshold of positivity. MENSA responses are in green and serum responses are in black.

Serial samples were collected at multiple time points in the interval 0 to 120 DPSO from eight of the twelve Newly Diagnosed Lyme Disease Patients. Examination of the temporal responses from the Newly Diagnosed Lyme Disease Patients identified at least three distinct patterns. The first pattern, "Non-responsive", was a failure to respond with detectable antibodies at all. One patient failed to respond at any time in serum or MENSA (patient IMD-EL-002, Fig 5A). The second response pattern, “Unresolved”, had positive MENSA and serum responses that remained elevated throughout the period of observation. The only patient in the Unresolved group (patient IMD-EL-004, Fig 5B) was admitted to the intensive care unit with neurologic complications that may have limited the success or consistency of treatment. Five of the six remaining patients presented typical or "Canonical" serological responses (Fig 5C–5G): serum anti-C6 and/or anti-pepC10 levels were elevated 14–28 DPSO and declined in the following months to substantially lower levels. In contrast, MENSA antibody levels started relatively high in most cases and then dropped below or near the baseline by 40 DPSO.

The most striking exception to these patterns was patient IMD-EL-012 (Fig 5H) who developed a dramatic anti-C6 response in the MENSA (anti-C6 > 4000 MFI-B at 16 and 42 DPSO) which remained strongly positive despite its evident decline at 100 DPSO, the last available time point. This patient also produced a modest serum anti-C6 response that declined to baseline by 42 DPSO. This patient’s anti-pepC10 MENSA response was similar to the other patients in both magnitude (~400 MFI-B) and duration (near baseline by 73 DPSO).

## Discussion

### MENSA-based diagnosis of Lyme disease infections is highly aligned with conventional serum-based diagnosis, but it is not identical

The objective of this study is to explore the utility of MENSA as an alternative and potentially complementary sample to serum for early diagnosis of Lyme disease. First, we demonstrate that the MicroB-plex Anti-C6/Anti-pepC10 Immunoassay is comparable in diagnostic performance to the CDC’s Two-Tier Lyme Immunoassay and to the commercially available Immunetics® C6 Lyme ELISA by testing each against the CDC Lyme Serum Panel I (Fig 2A and 2B and Table 1). Second, anticipating a requirement for greater analytic sensitivity for analysis of low concentration MENSA samples, we then show that the MicroB-plex Anti-C6/Anti-pepC10 Immunoassay has greater analytic sensitivity than the Immunetics® C6 Lyme ELISA using serial dilutions of a single positive serum sample (Fig 2C). Third, we examined 36 non-Lyme serum and MENSA Control samples drawn from patients in endemic and non-endemic areas to establish diagnostic C_0_ values (Fig 3 and S1 Fig). Fourth, in examination of MENSA and serum samples from real-world, Newly Diagnosed Lyme Disease Patients drawn 6–18 DPSO, diagnosis of ongoing Lyme infections was similar, but not identical, when examining serum and MENSA samples. Among the 12 Newly Diagnosed Lyme Disease Patients, six were positive and two were negative in both serum and MENSA samples; two were positive only in MENSA samples and two were positive only in serum samples (Fig 4 and Table 3). One non-endemic Control was positive for anti-C6 and anti-pepC10 antibodies in their serum sample, but not in the matching MENSA sample, possibly reflecting a past undiagnosed Lyme infection (S1 Fig and Table 3). Finally, when response patterns in serum and MENSA samples from eight Newly Diagnosed Lyme Disease Patients were tracked over periods as long as 120 days, multiple distinct patterns were observed. One patient generated no anti-C6 or anti-pepC10 in MENSA or serum throughout the period of observation; one patient produced substantial levels of serum and MENSA antibodies for at least 96 days, possibly indicating non-resolution of his infection; others (n = 5) displayed rises in both serum and MENSA antibody levels followed by declines to or near baseline; and one patient produced an unexpectedly large anti-C6 response in MENSA samples while producing modest levels of antibody in serum. Together, these observations suggest that MENSA-based diagnostics may improve the diagnostic sensitivity of blood-based testing and that it may also provide a measure for the success of therapy, at least in some patients.

### MENSA-based diagnostics can strengthen and complement pre-existing serum-based methods

Serum-based diagnostics for Lyme disease have been crucial to identify and track patients’ responses to infection. They have also been a source of concern because not all patients seroconvert. Here, we present a first step toward observing the active anti-*B*. *burgdorferi* humoral immune response using a new and fundamentally different sample matrix. Rather than measuring anti-*B*. *burgdorferi* antibodies in serum, we measure antibodies produced in vitro by circulating antibody-secreting cells (ASC) in a novel analytic fluid called MENSA. In contrast to serum antibodies, a complex mixture of past and ongoing immune responses, MENSA antibodies produced by ASC reflect only the active humoral response (Fig 1).

The abundance of antibodies measured in MENSA is several orders of magnitude lower than quantities typically present in serum. Consequently, it was promising that the levels of antibodies in MENSA were readily measured in eight of the twelve Newly Diagnosed Lyme Disease Patients. For the four patients with negative MENSA results, potential false negatives may arise due to inadequate MENSA preparation or the timing of sample collection especially if ASC production declines due to success of early antibiotic therapy, as in Fig 1.

In contrast, two patients had detectable *B*. *burgdorferi* antibodies in their MENSA samples while their corresponding serum samples were negative. One of these patients (IMD-EL-010) seroconverted a week after the time point included in Fig 4; the second (IMD-EL-011) was not observed to seroconvert in samples collected up to 25 DPSO. Because the emergence of ASC generally precedes the development of measurable serum titers (Fig 1), it is plausible that MENSA-based diagnostic tests may be superior earlier in the course of infection. The early diagnostic potential of MENSA in patients experiencing hospital-acquired *Clostridiodes difficile* infections depicts a similar trend [18]. The combination of serum and MENSA-based diagnostics may provide increased sensitivity without compromising specificity, compared to either sample type alone. In fact, the specificity of MENSA might be better than that of serum as evidenced by the seropositive, MENSA negative Control subject, indicating that serum may not adequately distinguish historical past infection antibodies from those of the current humoral response.

### MENSA responses may reveal clearance of *B*. *burgdorferi* infections

Another potential advantage of MENSA-based diagnostics is the ability to identify resolution of an infection by the reduction of circulating ASC to zero [12, 28]. Only one patient remained positive in both serum and MENSA samples to 96 DPSO (IMD-EL-004; Fig 5B). It is possible that this patient’s *B*. *burgdorferi* infection may not have been adequately resolved, putting them at risk of long-term sequelae. Unfortunately, follow-up information was not available. Among the other patients from whom samples were obtained, four out of six (IMD-EL-001, IMD-EL-003, IMD-EL-009, and IMD-EL-005) had MENSA values for anti-C6 and anti-pepC10 that declined to background at or before 40 DPSO and these declines were typically earlier than the positive measures in serum that lasted until and beyond 80 DPSO. However, two patients challenged this simple model: IMD-EL-008 (Fig 5C) had a low but sustained positive anti-C6 MENSA response until 100 DPSO; IMD-EL-012 (Fig 5H) had a substantially higher MENSA anti-C6 response that remained strongly positive to at least 100 DPSO. It is not known whether these patients resolved their infections.

### Limitations of this study

The work in this paper presents a new method to diagnose and track the status of ongoing *B*. *burgdorferi* infections. There are multiple limitations of the presented work that frame crucial questions for subsequent examination. Only twelve predominantly white, male and middle-aged patients were enrolled in this preliminary study, and those twelve were from a relatively small geographic area. Future work will attempt to include greater patient diversity and geographical distribution.

A second limitation was the focus on just two (C6 and pepC10) of the numerous Lyme-specific antigens that could provide additional diagnostic information. Even though anti-pepC10 added very little to the sensitivity of anti-C6, multiple investigators have illustrated the potential for the measurement of patient antibody responses to other *B*. *burgdorferi*-specific antigens [29–31], and we plan to explore more of these responses in the future.

A third limitation is that we used a combined IgM and IgG detection cocktail to be comparable to the Immunetics® C6 Lyme ELISA™, so it is unclear which is the predominant isotype in the positive MENSA and serum samples assessed here. In future studies, it would be helpful to measure IgG and IgM separately and perhaps add other isotypes.

Fourth, the time window for sample collection was not carefully designed in this study; even for "early Lyme", it tended toward later (6–28 DPSO) samples in order to enhance the likelihood of positive responses in unproven MENSA-based diagnostics. Sampling earlier in the course of infection may provide a better view of rising host responses when ASC emerge into the blood. In addition, this study was not designed to provide detailed clinical follow-up, which could help us better understand infection resolution and prolonged MENSA responses.

### Future studies of MENSA in Lyme disease diagnostics

For early diagnosis of Lyme disease this preliminary study illustrates that MENSA-based diagnostics may complement conventional serologic diagnostics. In addition, MENSA-based diagnostics may identify patients who have successfully resolved their *B*. *burgdorferi* infections earlier and more clearly than serum-based measurements. Future studies of the potential clinical utility of MENSA for diagnosis and tracking of Lyme disease may require additional antigens, measurement of different Ig isotypes, and more patients examined over a longer time frame and from a larger geographic area.

The MENSA-based diagnostic approach may enable the earlier diagnosis of Lyme infections, while offering an earlier measure of disease resolution. Furthermore, MENSA-based immunoassays have the potential for diagnosis of recurrence or reinfection in highly endemic areas, particularly among patients with high levels of serum antibodies against prior *B*. *burgdorferi* infections.

## Supporting information

S1 Fig Antibodies specific for C6 and pepC10 are present in serum and MENSA samples from Newly Diagnosed Lyme Disease Patients and absent in samples from Control subjects. To determine clinical cut-off (C_0_) values, the MicroB-plex Anti-C6/anti-pepC10 Immunoassay was tested against samples collected from non-Lyme Controls who lived in Non-endemic (n = 31) and Endemic (n = 5) regions. Samples from two Newly Diagnosed Lyme Disease Patients were included as positive controls (Acute Lyme, n = 2). C_0_ values are indicated by dashed red lines. All samples were measured in duplicate and the mean value is displayed. A single "Control" serum sample was positive for both anti-C6 and anti-pepC10 and it exceeded the median value by greater than 10-fold; it was excluded from the calculation of the C_0_, shown here as the single gray dot above the red dashed lines in A and B.
(PDF)
