# Supplementary material for: Circulating antibody-secreting cells are a biomarker for early diagnosis in patients with Lyme disease
Source: PLoS One. 2023 Nov 3;18(11):e0293203. doi: 10.1371/journal.pone.0293203 (PMC10624293; doi:10.1371/journal.pone.0293203)
Supplement: S1 Fig — To determine clinical cut-off (C0) values, the MicroB-plex Anti-C6/anti-pepC10 Immunoassay was tested against samples collected from non-Lyme Controls who lived in Non-endemic (n = 31) and Endemic (n = 5) regions. Samples from two Newly Diagnosed Lyme Disease Patients were included as positive controls (Acute Lyme, n = 2). C0 values are indicated by dashed red lines. All samples were measured in duplicate and the mean value is displayed. A single "Control" serum sample was positive for both anti-C6 and anti-pepC10 and it exceeded the median value by greater than 10-fold; it was excluded from the calculation of the C0, shown here as the single gray dot above the red dashed lines in A and B. (PDF) [file pone.0293203.s001.pdf]

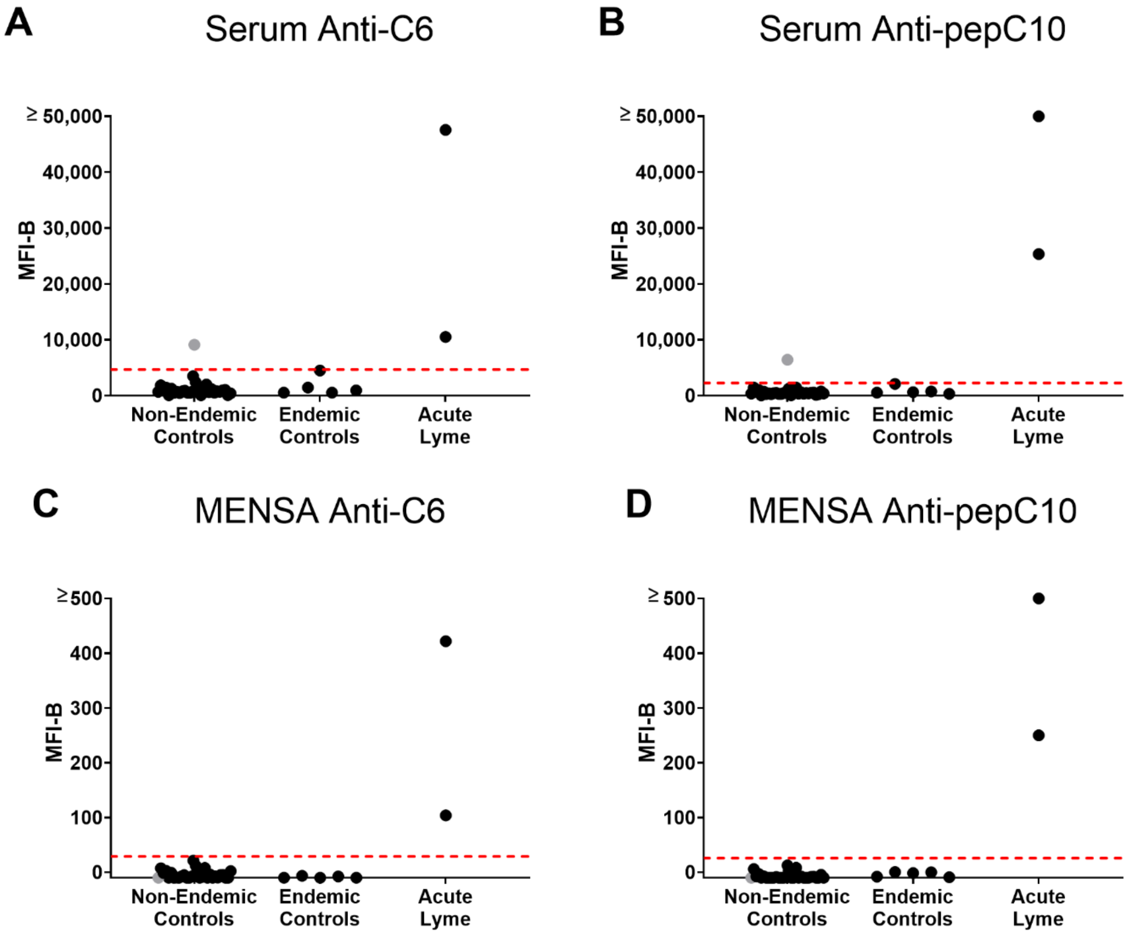

S1 Fig. Antibodies specific for C6 and pepC10 are present in serum and MENSA samples from Newly Diagnosed Lyme Disease Patients and absent in samples from Control subjects. To determine clinical cut-off (C_0) values, the MicroB-plex Anti-C6/anti-pepC10 Immunoassay was tested against samples collected from non-Lyme Controls who lived in Non-endemic (n=31) and Endemic (n=5) regions. Samples from two Newly Diagnosed Lyme Disease Patients were included as positive controls (Acute Lyme, n=2). C_0 values are indicated by dashed red lines. All samples were measured in duplicate and the mean value is displayed. A single "Control" serum sample was positive for both anti-C6 and anti-pepC10 and it exceeded the median value by greater than 10-fold; it was excluded from the calculation of the C_0, shown here as the single gray dot above the red dashed lines in A and B.
